# American Academy of Dental Science

**Published:** 1870-11

**Authors:** Edward N. Harris


### AMERICAN ACADEMY OF DENTAL SCIENCE.

The third annual meeting of the American Academy of Dental Science was held in Mercantile Hall, Boston, September 26, 1870, at 12 o'clock, M.

The president, Dr. Daniel Harwood, on taking the chair, congratulated the members upon the present prosperous condition of the Academy, and upon the encouraging prospects for the future.

There was a good attendance of members present, including a number from other States, among whom were Dr. J. H. Foster, of New York, Dr. F. N. Seabury, of Providence, R. I., Dr. Elbridge Bacon, of Portland, Maine, Dr. C. K. Fiske, of St. John, New Brunswick, and Dr. Edward Gage, of Paris.

The following officers were elected for the ensuing year:

President—Daniel Harwood, M. D.

Vice President—E. T. Wilson, M. D.

Corresponding and Recording Secretary—E. N. Harris, D. D. S.

Treasurer—E. G. Tucker, M. D.

Librarian—John Clough, M. D.

Board of Censors—Drs. E. G. Tucker, J. L. Williams and W. W. Codman.

At this and the previous monthly meeting, the following new members were admitted:

Drs. T. B. Hitchcock, L. D. Shepard and T. H. Chandler, professors in the Harvard Dental College, Boston, were elected Active Fellows; also Drs. J. T. Codman, of Boston, J. K. Batchelder, of Salem, and S. P. Martin, of Worcestor.

Drs. F. N. Seabury, T. D. Thompson, M. B. Mead, of Providence, R. I., James McManus, of Hartford, Conn., and H. M. Bowker, of Montreal, Canada, were elected Associate Fellows.

Dr. Amos Westcott, of Syracuse, N. Y., was elected an Honary Fellow.

The annual address was delivered by Joseph H. Foster, M. D., of New York, on the subject of "Professional Responsibilities." Dr. Foster occupied one hour and twenty minutes in the delivery of his address, which was a very able production, eloquently delivered, and received the hearty applause of the assembly.

The thanks of the Academy were presented to Dr. Foster, and a copy of his address requested for publication and for preservation in the archives of the society.

Interesting papers were read by H. F. Bishop, D. D. S., of Worcstor, on "Dental Societies; by John Clough, M. D., of Woburn, on "Causes of Diseased Teeth and Gums, and Preventive Measures for their Preservation;" and by Dr. Joseph E. Fisk, of Salem, on "The Manufacture of Mineral Teeth."

At half past 5, P. M. the Academy adjourned to the Parker House to partake of the Third Anniversary Dinner, which was a very elegant affair, served in Parker's usual elegant style, and the festivities were continued until a late hour.

EDWARD N. HARRIS, D. D. S.
*Cor. and Rec. Secretary.*

